# Novel Digital Architecture of a “Low Carb Program” for Initiating and Maintaining Long-Term Sustainable Health-Promoting Behavior Change in Patients with Type 2 Diabetes

**DOI:** 10.2196/15030

**Published:** 2020-03-04

**Authors:** Charlotte Summers, Kristina Curtis

**Affiliations:** 1 Diabetes Digital Media Coventry United Kingdom; 2 Faculty Research Centre for Advances in Behavioural Science University of Coventry Coventry United Kingdom

**Keywords:** type 2 diabetes, behaviour change, nutrition, digital intervention, low carb, type 2 diabetes remission, eHealth

## Abstract

Globally, the burden of noncommunicable diseases such as type 2 diabetes is crippling health care systems. Type 2 diabetes, a disease linked with obesity, affects 1 in every 30 people today and is expected to affect 1 in 10 people by 2030. Current provisions are struggling to manage the trajectory of type 2 diabetes prevalence. Offline, face-to-face education for patients with type 2 diabetes has shown to lack long-term impact or the capacity for widespread democratized adoption. Digitally delivered interventions have been developed for patients with type 2 diabetes, and the evidence shows that some interventions provide the capacity to support hyperpersonalization and real-time continuous support to patients, which can result in significant engagement and health outcomes. However, digital health app engagement is notoriously difficult to achieve. This paper reviews the digital behavior change architecture of the Low Carb Program and the application of health behavioral theory underpinning its development and use in scaling novel methods of engaging the population with type 2 diabetes and supporting long-term behavior change.

## Background

The prevalence of both prediabetes and type 2 diabetes is increasing globally. Currently, 4 million people are diagnosed with diabetes in the United Kingdom, 90% of whom have type 2 diabetes. By 2030, nearly 10% of the UK adult population may require diabetes treatment [1]. In terms of health inequality, diabetes more commonly affects people of low (rather than high) socioeconomic status, particularly women [2,3]. By 2035, the UK National Health Service (NHS) is predicted to spend approximately £17 billion a year on treatment for diabetes and avoidable diabetes-related complications, which is approximately 17% of its entire budget [4].

Patients diagnosed with type 2 diabetes are known to have difficulties adhering to their treatments (medications, diet, and lifestyle change) [5,6], and despite many recent technical breakthroughs in health care, human behavior remains the largest source of variance in health-related outcomes [7]. Nonadherence to treatment negatively affects NHS treatment efficacy and finances [8-11]. Nonadherent patients diagnosed with type 2 diabetes are more likely to have higher blood glucose levels (hyperglycemia), resulting in higher hemoglobin A_1c_ (HbA_1c_) levels [12]. Previous prospective studies in patients with type 2 diabetes have shown an association between the degree of hyperglycemia and increased incidence and progression of microvascular complications (diabetic retinopathy, loss of vision, and nephropathy) [13,14], sensory neuropathy [13,15], myocardial infarction (heart attack) [13,16,17], stroke [18], macrovascular mortality [19-21], and all-cause mortality [20,22-25].

In the UK Prospective Diabetes Study, a 1% reduction in the average HbA_1c_ level was associated with a 21% reduced risk of any adverse outcome related to diabetes, 21% reduced risk for deaths related to diabetes, 14% reduced risk for myocardial infarction, and 37% reduced risk for microvascular complications [26].

Research has shown that having better glycemic control is associated with a better quality of life [27]. Psychosocial factors often determine self‐management behaviors and the ability to adhere to treatment. Psychosocial variables (such as depression) are often strong predictors of medical outcomes such as hospitalization [28]. The American Diabetes Association released a position statement; the first recommendation stated that psychosocial care should be integrated with collaborative, patient-centered medical care and provided to all people with diabetes, with the goals of optimizing health outcomes and health-related quality of life [29].

## Patients’ Behavior

Patients’ behavior directly contributes to their treatment success, with doctors relying on patients to take their prescribed medication alongside making and maintaining dietary and lifestyle changes. Many of the most significant challenges in health care, specifically in long-term or chronic conditions, such as type 2 diabetes, will only be resolved if we can influence behavior and support sustainable behavior change.

An analysis from a secondary care diabetes clinic in the United Kingdom found that 86% of those with type 2 diabetes are overweight or obese. Obesity is associated with significantly worse cardiovascular risk factors, suggesting that more active interventions to control weight gain would be appropriate to help address the increasing burden of obesity and type 2 diabetes on the NHS. The National Institute for Health and Care Excellence (NICE) guidelines established that adults with type 2 diabetes who are overweight, should be set an initial body weight loss target of 5%–10%. [30] Regardless of the interventions used to lose weight—pharmacological [31] or behavioral [32]—the weight is commonly regained [33]. Typically, half the weight lost is regained in the first year. Weight regain often continues up to 3-5 years after treatment and, on average, 80% of people return to or exceed their preintervention weight [34]. Similarly, relapse rates are high for individuals who initiate attempts to stop smoking [35,36] and those who try to reduce alcohol consumption [37]. Therefore, effective interventions that consider known factors associated not only with initial weight loss but also critically with weight loss maintenance such as building on internal motivations to lose weight, establishing social support mechanisms, identifying coping strategies, or providing support for self-efficacy and autonomy can all enhance weight loss maintenance, which is crucial for the long-term success of any weight loss interventions [38].

There is considerable evidence that health behaviors can be effectively modified through behavior change interventions [39-42]. However, there is a disproportionate number of behavior change theories in the academic literature, including both those that assess the use of interventions for health behavior initiation and those that theorize interventions essential to behavior change maintenance [43]. In addition, behavior change theory is most frequently used to explain behavior itself rather than potential behavioral change interventions [44].

## Novel Application of Behavior Change Theory

This paper introduces the Low Carb Program Health Behaviour Change platform—a digital architecture developed to initiate and maintain behavior change in patients with type 2 diabetes and other chronic metabolic health conditions.

The purpose of this paper is to explore the conceptual hypotheses and theories around which the digital architecture has been built, with the aim of contributing to current psychological literature, simulating research, and encouraging the development of new digital applications created with the intention of initiating and maintaining health-related behavior change.

The Low Carb Program is a digitally delivered, automated, structured health intervention for adults, personalized to people with type 2 diabetes, prediabetes, and obesity. User data are used to personalize the experience member’s receive. The use of user data has been suggested to improve patient engagement through individualization of the participant’s experience [45].

In the on boarding of the program, patients are instructed to select a health goal and input their current health status and demographics including age, gender, ethnicity, and dietary preferences—all of which are used to personalize the participant’s experience of the platform.

Participants are given access to therapeutic nutrition education modules. Education is personalized to the user’s health status, age, ethnicity, and dietary preferences. A new module is available each week over the course of 12 weeks. Lessons are taught through videos, written content, or podcasts of varying lengths (approximately 3-12 minutes long). The program encourages participants to make behavior changes based on “Action Points” or behavior-change goals at the end of each education module.

Participant’s health goals are supported with behavior change resources that are available to download including information sheets, meal plans, and suggested food substitution ideas.

Users are matched within the platform to a digital buddy and are given access to a peer-support forum available 24 hours a day. Behavior change is maintained through continual engagement, new modules, and nudges to track health outcomes and interact with the support community.

Automated feedback and nudges are provided to users based on their use of the program through emails and native in-app push notifications, and participants are notified when the next week’s module is available.

## Outcomes of the Platform in the Real World

The 1-year outcomes of the Low Carb Program, which utilizes the behavior change architecture, were previously published [46]. The 1-year outcomes for people with type 2 diabetes were reported in a single-arm longitudinal study that assessed users engagement within the platform as well as their health outcomes including weight, HbA_1c_ levels, and medication dependency.

Participants who completed the program lost an average of 7% body weight and reduced their HbA_1c_ levels by 1.2%; in addition, 40% eliminated a diabetes medication from their treatment. Further, 26% of participants completing the program were classified as being in remission from type 2 diabetes at 1 year. The platform also demonstrated a 71% retention at 1 year.

The results were collected after a year of the individual joining the platform, indicating that the behavior change wheel is also of clinical importance for maintaining positive health behaviors acquired during the initiation period.

The Low Carb Program, launched in November 2015, is available as an iOS, Android, and Web app and has been downloaded over 425,000 times. It includes digital tools for submitting self-monitoring data on a number of different variables including blood glucose levels, blood pressure, mood, sleep, food intake, activity, medication consumption, and body weight. The program is integrated with wearable and Bluetooth-enabled devices. As such, data can also be brought into the platform without requiring user input (Multimedia Appendix 1 and 2).

## The Capability, Opportunity, Motivation, and Behavior Model of Behavior Change and Low Carb Program

### Overview

The COM-B (capability, opportunity, motivation, and behavior) model was developed as a response to the inability of the majority of prevailing theories to provide strategies to change behavior and as part of a “method for characterizing interventions and linking them to an analysis of the targeted behavior” [47]. It is essentially a behavioral system that posits the interaction of three components—capability, opportunity, and motivation—which result in the performance of behavior [48]. COM-B canvases a range of mechanisms involved in behavior change and is “intended to be comprehensive, parsimonious and applicable to all behaviours” [48].

Each component can be subdivided into two heuristics: capability can be either “psychological” (involving knowledge and psychological skills) or “physical” (involving physical skills); opportunity can be either “social” (involving social influences and cultural norms) or “physical” (involving environmental resources, triggers, time, locations, and physical barriers); motivation can be either “reflective” (involving conscious planning or evaluation) or “automatic” (involving emotional responses, impulses, and reflexive responses) [47].

The following section will map each feature within the Low Carb Program to the relevant COM-B domain.

### Social Opportunity

#### Peer Support Feature

Social opportunity refers to the people’s environment that either hinders or facilitates their behavior [49]. Social influences can be defined as “interpersonal processes that can cause individuals to change their thoughts, feelings, or behaviours” and includes constructs such as social norms, social comparisons, modelling, social support, and social pressure [50].

Social relationships are adaptive and crucial for survival. Social connections have powerful influences on health and longevity. Lacking social connection qualifies as a risk factor for premature mortality [51].

Social support has received attention as a mediator or moderator of health outcomes [52]. Social support has been facilitated in behavior change interventions in distinctive approaches in diabetes education. Researchers have examined the impact of group-based training [53,54]; peer group support that included telephone calls [55,56]; organized internet peer group forums with and without the addition of personal coach support [57,58]; and support from peers, spouse, family, and friends [59].

An empirical study of knowledge creation and social support on a diabetes online community forum concluded that being a member of the community forum had a positive impact on its members’ wellbeing and can help members manage their relationship with health care professionals. The authors concluded that members felt less emotionally burdened while managing their diabetes as a result of being a member of the community [57].

In an overview of peer support models to improve diabetes self-management and clinical outcomes, interventions that facilitate peer support are found to be a low-cost approach to encouraging dietary changes both in weight and diabetes managements [60,61]. Social networking and publicly sharing your progress on social media has been shown to be a beneficial and effective strategy for weight loss [62,63]. The Low Carb Program accommodates a dedicated peer support community forum. Patients are able to access the forum 24/7, providing users a dynamic social network that allows real-time interactions with their peers on a continuous basis. This facilitates a constant source of information, knowledge, personal anecdotes, and behavioral reinforcement from their peers worldwide. Users are encouraged to ask questions and share their goals and progress via facilitated discussions such as “Weigh in Wednesday” threads. Users of the Low Carb Program have access to the social support forum even after they have completed all the education modules. It is hypothesized that a significant proportion of the success of the Low Carb Program could be attributed to the forum, even users who do not actively post are able to “lurk,” meaning that they regularly read threads but do not necessarily comment or actively engage with the content.

#### Buddy System Feature

The Low Carb Program seeks to facilitate the use of a social support network in a digital setting by partnering up new members with existing users who have successfully completed the intervention, providing each user with a digital “buddy.” Members are matched on a number of attributes, including self-selected health goals, demographics including age and ethnicity, diabetes type, and starting medication regime. Buddies facilitate observational learning in a digital setting, “communicating” with the new members via emails and in-app push notifications. The “social opportunity” element of the behavior change wheel asserts that people can witness and observe a behavior conducted by others and then reproduce those actions. If individuals see successful demonstration of a behavior, they are also likely to complete the behavior successfully.

Research on the effectiveness of a buddy system in other digital settings is somewhat contradictory, particularly when analyzing different age groups. Sylvetsky et al [64] found that assigning young, healthy, and motivated volunteer partners or “buddies” to adolescents with type 2 diabetes did not result in an improvement of HbA_1c_ levels; however, this was not the case for adults with type 2 diabetes, where “buddying up” resulted in an effective improvement of HbA_1c_ levels. The latter findings were also observed by Greaney et al [65]: Individuals paired with a buddy who offered support showed greater reduction in multiple risk behaviors compared to nonpaired controls. This research suggests that engagement with individuals that share similar conditions and demographics could enhance goal attainment and result in more desirable health outcomes.

### Reflective Motivation

#### Goal Setting Feature

Reflective motivation involves our conscious and reflective processes that motivate our behavior [47] and includes goal setting. Goals represent an individual’s goals to achieve personal self-change, enhanced meaning, and purpose in life [66]. Evidence suggests that goal setting can act as an effective behavioral treatment strategy to change health behaviors and improve adherence to lifestyle intervention programs, such as diabetes management [63] and obesity prevention [67]. To enhance engagement and adherence to behavior change interventions in adults with obesity, goal setting has been suggested to be essential in the improvement of health outcomes [67].

The Low Carb Program provides patients with the opportunity to self-select their goals for the platform. Beyond simply setting a goal, the “Crystal Ball Technique” [68] is used, whereby members are nudged to consider a future reality in which their goal has been achieved; they are asked to think about what achieving their goal would mean to them and draw on their social norms to share who they think will notice if they are to be successful in attaining their goal. A systematic qualitative review of effectiveness of solution-focused therapy found that 74% of studies reported significant positive benefit from this solution-focused therapy [69]. Motivational solution-focused therapy has been previously utilized to encourage entry into an intervention intended to improve glycemic control in young people with poorly controlled type 1 diabetes. The researchers found that the approach produced a significant improvement of 1.5% in HbA_1c_ levels, concluding that a solution-focused group intervention is promising for improving HbA_1c_ levels [70]. Locke and Loatham [71] developed the theory of goal setting and theorized that in order for a goal to be motivating, it needs to be specific and challenging; it also requires commitment, feedback, and task complexity [71].

When setting a goal within the platform, users are nudged to reflect on how close they perceive themselves to be to achieve their goal using a sliding scale of 1 and 10 points. Periodically, as they are using the platform, they are prompted to “check-in” with their initial goal and report on the same scale.

When it comes to maintaining behavior change, a systematic review of the psychosocial and sociodemographic determinants of physical activity maintenance [72] revealed that maintainers had higher self-efficacy and intention compared with those who relapse. Therefore, beliefs about capabilities, motivations, and goals may be among the strongest variables associated with behavior change maintenance. Additionally, a motivation-focused weight loss maintenance program is an effective alternative to a skill-based approach [73]. The combined research on goal setting across many different contexts and fields of study demonstrates that goal setting encourages a person to try harder and for longer periods of time, with less distraction from the task at hand [74] and therefore is rightfully integral to the Low Carb Program.

### Psychological Capability

#### Health Tracking Feature

Psychological capability refers to people’s physical psychological skills, for example, knowledge, strength, or stamina to engage in mental processes [49]. Included in this domain is “behavioural regulation” defined as “anything aimed at managing or changing objectively observed or measured actions” and includes constructs such as self-monitoring, action planning, and habit breaking [50].

Monitoring goal progress is an effective self-regulation strategy that promotes goal attainment, as it serves to identify discrepancies between the current state and the desired state and thus enables people to recognize when additional effort or self-control is needed. Interventions that increase the frequency of progress monitoring are likely to promote behavior change [75].

According to literature reviews, in addition to setting a goal to promote behavior change, tracking its progress is just as crucial and effective to promote sustained behavior change [63]. Recent findings suggest that program interventions that elevate the frequency of progress monitoring are likely to induce behavior change [75]. Among the several benefits of self-tracking and reviewing tracked data are the following: patients can identify trends and correlations from their data and become more independent in managing their conditions; tracking can also provide opportunities for patient education [76].

The Low Carb Program offers an integrated tracking mechanism whereby patients can self-track their weight, food, mood, blood glucose levels, medications, sleep, blood pressure changes, cholesterol levels, insulin levels, and ketone levels. The platform also has many wearable devices and Bluetooth-enabled devices such as blood glucose meters or weighing scales, with which users can bring in data from devices to monitor trends and view interactions with other variables they may be tracking. The platform also nudges patients to embrace novel methods of tracking progress, for example, taking selfies, from which there are machine learning algorithms that can predict waist-to-hip ratios. The Low Carb Program reinforces behavior change by providing intelligent insights based on the tracked data into trends. The platform then nudges users when their tracked data are congruent with the trends required for their self-selected goal attainment.

#### Memory Aids and User-Engagement

**Figure 1:**
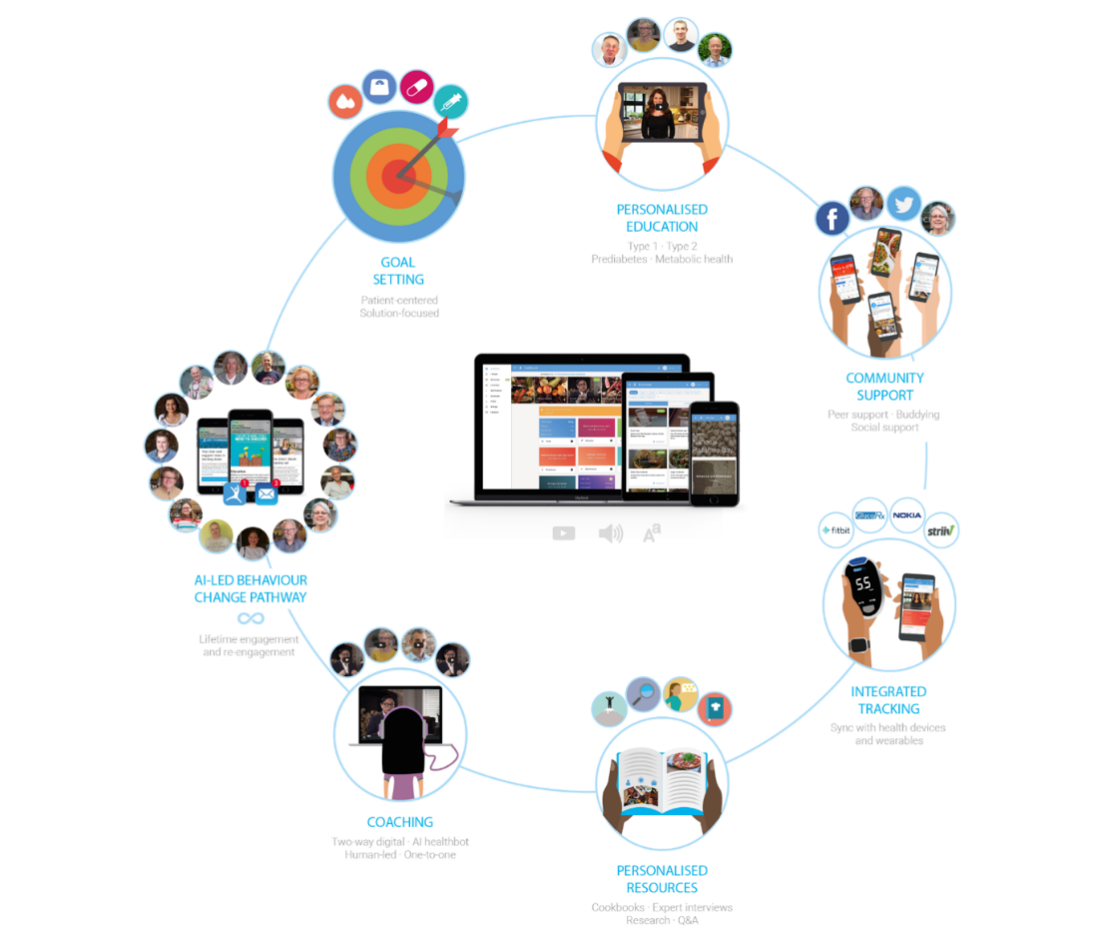
Low Carb Program Behaviour Change platform architecture.

One reason that behavioral change interventions do not deliver sustained effects is that they do not consider unintentional reasons for patients failing to adhere to their treatment plan. “Simply forgetting” is an example of unintentional nonadherence and serves as the most commonly reported reason for people not taking their medication [77-79]. Recent trials have demonstrated the benefits of telephone interventions to remind patients to pick up new prescriptions and talk about adherence [80,81]. However, utilizing staff to telephone patients is often cost prohibitive. Short message service or text message reminders are a less expensive way forward [82]. Research suggests that reminders can significantly increase patient attendance to clinic appointments [83] and reduce no-shows across health care settings. A recent paper showed that sending multiple notifications could improve attendance and text notifications improved attendance [84]. A text messaging support system was also shown to improve self‐efficacy and adherence, engaging a classically difficult-to-reach group of young people [85]. Texting messages has proven to be a productive communication method for promoting behaviors that support weight loss in overweight adults [86]. Unfortunately, text-message interventions are difficult to implement in organizations that do not have a large-scale text-message distributor. For these reasons, a richer and more comprehensive set of behavior change techniques and technology-based interventions should be explored. The Low Carb Program architecture (Figure 1) utilizes email and in-app push notifications to encourage user’s continual engagement with the program. Users receive notifications when a new module is added or opened with that week’s “actions.” When they have a new reply from a member of the community, they also receive nudges to continue tracking their progress and when feedback is provided, for example, a new insight is generated from their tracked data.

The language used within these notifications and nudges is also considered, building on Locke’s Goal Setting Theory [87]: Telling someone to “Try hard” or “Do your best” is less effective than “Try to get more than 80% correct” or “Concentrate on beating your best time.” The Low Carb Program behavior change architecture encourages health promoting behavior such as “Try to log more hours of sleep” when they are getting less than the recommended amount or “Continue your great blood glucose streak today, track your readings and stay within your targets.” These notifications have been tested within the architecture and optimized for different users within the behavior change programs.

In addition to the emails and push notifications running alongside the initial implementation phases of the education programs, notifications and emails are used to re-engage users who have not maintained their engagement within the programs. Users are nudged back into the program with tailored messages based on demographics, time elapsed, and their self-selected goal.

### Automatic Features

#### Personalized Content Contextualized Within Cultural Norms

Research has previously demonstrated that gender, attitudes, subjective norms, perceived pros, different self-efficacy expectations, and habit strength are significantly associated with healthy eating habits [88]. The NICE guidelines in the United Kingdom actually state that information should be provided in an accessible format (particularly for people with physical, sensory, or learning disabilities and those who do not speak or read English) and educational materials should be translated, if needed [89]. The general consensus from the behavior change literature is that tailored interventions, which address an individual’s specific circumstances and concerns, should be more likely to stimulate change than untailored interventions. Studies have found that compared to untailored messages, tailored messages are more likely to be read and remembered; saved; discussed with others and perceived by readers as interesting, personally relevant, and written especially for them [90-93]. In addition, culturally tailored education, health-promoting information, and guidance to ethnic/linguistic minority groups has found to significantly improve their risk factors for progression to diabetes such as excessive weight and obesity [94] as well as glycemic control and diabetes knowledge compared to nonculturally tailored content [95-97]. Across several economically developed countries, including the United Kingdom, a number of ethnic groups experience higher levels of morbidity and mortality compared to the majority of the white European-origin population. Thus, creating culturally tailored health-promoting approaches is essential to improve health outcomes in people affected by diseases such as diabetes [97].

However, some literature highlights that although tailoring information delivered as part of behavior change interventions is a proven approach to enhancing message applicability, it is not the only approach to do so, and under many circumstances, it may not be the preferred choice, with some researchers citing insufficient evidence on the clinical effectiveness or cost-effectiveness of these adapted approaches [96,98]. This is where the Low Carb Program behavior change architecture may be advantageous over other more traditional methods of education delivery. As a Web and mobile behavior change platform, the education can be tailored as an individual signs up to the program with intelligent coding used to determine the tailored information that users subsequently see; this includes personalized education video modules delivered in native language and tailored to cultural norms determined by users’ ethnicity and language preferences, modified meal plans, and recipes tailored to dietary preferences and tailored content within the “lifestyle” area of the app based on their self-selected goal, age, and gender. The onboarding process also assigns the users a virtual buddy based on a “best fit” criteria, matching previous program completers as far as possible to the user’s gender, age, and disease profile and starting a medication regime and self-selected goal, increasing the perceived personal relevance and applicability of the information received within the behavior change intervention.

#### Incremental Stages of Change

Key recommendations from leading experts in the field of behavior change advise to start with small behavioral changes and build upon these incrementally [49]. In addition, insights from goal setting theories that support sustainable behavior change show that deadlines at stages need to be set, and they need to apply an appropriate amount of pressure while still being achievable [71]. The Low Carb Program architecture has a number of elements to support incremental changes. These stages of change do not exclusively facilitate the five transtheoretical model stages of change, but support change, aggregating over time to establish sustainable health-promoting behavior. The education modules are unlocked on a weekly basis, encouraging incremental behavior changes over time. Each education module is supported with an “action points” video, outlining suggested changes to make over the subsequent 7 days before the next module opens; these are precise actions with a set deadline by which the behavior modifications are to be made. By delivering the education in this way, the user will not be overwhelmed with lifestyle changes and instead, build on them week by week. The user also gets the opportunity to pause and restart their program at any time, closing opened modules and restarting their journey to take account of circumstances that may be impeding their ability to succeed.

#### Web-Based and Mobile-Based Delivery of Information

In order to address the growing burden of type 2 diabetes, prediabetes, and obesity as well as other chronic conditions, the promotion of wellbeing and behavior change interventions requires the delivery of scalable, engaging, and effective interventions aimed at sustainable behavioral change. The internet and pervasiveness of mobile devices offers an opportunity to reach this goal.

Research demonstrates that smartphone or Web apps offer significant benefits for patients in terms of patient care, education, and promoting behavior change, although the impact on several aspects of Web and mobile health delivery have not been clear, such as the cost-effectiveness and the adequacy of the infrastructure [99]. Burner et al [100] suggest that mobile health is a promising approach to support patients with diabetes and their health outcomes, and others [101] suggested that the integration of mobile apps with diabetes management can be beneficial for the lifestyle of the patients by providing useful health and nutritional information. However, research points to the need of further studies to be undertaken to establish the effectiveness of in-person delivery compared to Web-based delivery of behavioral change programs [102].

Internet-based interventions have been utilized with success in behavior change interventions promoting mental fitness [103] and to deliver cognitive behavioral therapy for people experiencing symptoms of depression and anxiety [104]. One of the major advantages of internet-based interventions is their scalability, as they are able to engage hard-to-reach individuals and can reduce the cost of care by reducing therapists’ time [105].

From a diabetes perspective, technology-enabled diabetes self-management solutions significantly improve blood sugar levels (usually, these effects are observed at 3-12 months). The evidence from this systematic review indicates that organizations, policy makers, and health care payers should consider integrating these solutions in the design of diabetes self-management education and support services. In conclusion, digital (mobile phone) health solutions that incorporate evidence-based, behaviorally designed interventions can improve access to diabetes self-management education and ongoing support [106].

A meta-analysis of 13 studies including 6 randomized controlled trials found that there were statistically significant reductions of HbA_1c_ levels in the intervention groups at the end of the studies. The researchers concluded that beyond improving HbA_1c_ levels in patients with diabetes, the use of apps reinforces the perception of self-care by contributing better information and health education to patients. Patients also become more self-confident in their ability to manage their diabetes, mainly by reducing their fear of not knowing how to deal with potential hypoglycemia episodes that may occur [107]. One interesting observation of the researchers was from an exploratory subgroup analysis, which showed that having a clinical decision-making function in app-based interventions was not associated with a greater HbA_1c_ reduction. This implies that the value of the app-based delivery of behavior change may be in the self-efficacy generated by the patients themselves using the app rather than its use as a replacement of their clinical reviews with their own health care professional teams.

The use of the Low Carb Program digital platform was never designed or intended to replace the intricate relationship between patient and health care professionals. Instead, it serves to augment or assist usual care, for instance, support behavior and lifestyle changes, which doctors feel inadequately trained to counsel patients on [108,109], despite the fact that the NICE guidelines specify giving lifestyle advice as a first-line intervention for diabetes, obesity, and high cholesterol levels.

### Conclusions

The prevalence of obesity and subsequent noncommunicable, metabolic conditions such as type 2 diabetes, polycystic ovarian syndrome, Alzheimer Disease, and some cancers is drastically increasing. Patients diagnosed with diabetes have problems adhering to their treatments including medications and lifestyle change. Current health care systems are struggling to provide adequate training and education provisions necessary to empower patients to adequately self-manage their conditions. Patients’ behavior contributes significantly to their treatment success, which implies the necessity for behavioral solutions to achieve long-term sustainable change. However, there still remains uncertainty over how long the behavior change effects last and the optimal methods of delivery, in particular, the intricate interactions of program characteristics required to support sustainable change.

The key elements that make up the Low Carb Program are grounded in the COM-B model and evidence-based behavior change techniques that are shown to be effective in digital platforms for behavior change interventions that support weight loss, increase physical activity, and improve self-efficacy of chronic disease management.

The Low Carb Program is an effective tool to help support the initiation and maintenance of health promoting behavior in people with type 2 diabetes, as demonstrated by industry-leading health and engagement outcomes of education delivered using the platform architecture. There is a clear requirement for programs to be utilized as an adjunct to the current care pathways for people with chronic conditions and obesity. This poses further research questions, such as how digital interventions can be used within a blended model of health care and other long-term health conditions.

Regarding the Low Carb Program, further research is required to systematically test the different elements of the ecosystem for their influence on both engagement and sustainable change. For instance, it may be the case that certain individuals require access to a peer support community to see long-term change and health outcomes, while others may require access to data to see real-time feedback to reinforce behaviors. Due to the size of the population within the platform, there is also an opportunity to understand how to improve the effectiveness of support for patients to achieve and maintain remission.

Research is required to explore the biological and psychological characteristics, online social engagement, interactions, and social context of patients with type 2 diabetes who use the digital platform and achieve type 2 diabetes remission and maintenance compared to patients who do not. This could be used to develop risk stratification models that can be applied to effectively triage patients and identify the targeted support they need to achieve and maintain type 2 diabetes remission as well as further hyperpersonalize the behavior change ecosystem.

## Appendix

Multimedia Appendix 1 Low Carb Program apps on desktop, iOS, and Apple Watch.

Multimedia Appendix 2 App screenshot: home.

## Abbreviations

COM-B: capability, opportunity, motivation, and behavior
NHS: National Health Service
NICE: National Institute for Health and Care Excellence

## References

[1] Gatineau M, Hancock C, Holman N, Outhwaite H, Oldridge L, Christie AL Public Health England.

[2] Espelt A, Borrell C, Roskam AJ, Rodríguez-Sanz M, Stirbu I, Dalmau-Bueno A, Regidor E, Bopp M, Martikainen P, Leinsalu M, Artnik B, Rychtarikova J, Kalediene R, Dzurova D, Mackenbach J, Kunst AE (2008). Socioeconomic inequalities in diabetes mellitus across Europe at the beginning of the 21st century. Diabetologia.

[3] Imkampe AK, Gulliford MC (2011). Increasing socio-economic inequality in type 2 diabetes prevalence--repeated cross-sectional surveys in England 1994-2006. Eur J Public Health.

[4] Hex N, Bartlett C, Wright D, Taylor M, Varley D (2012). Estimating the current and future costs of Type 1 and Type 2 diabetes in the UK, including direct health costs and indirect societal and productivity costs. Diabet Med.

[5] Capoccia K, Odegard PS, Letassy N (2015). Medication Adherence With Diabetes Medication. Diabetes Educ.

[6] Gadkari AS, McHorney CA (2012). Unintentional non-adherence to chronic prescription medications: How unintentional is it really?. BMC Health Serv Res.

[7] Schroeder SA (2007). We Can Do Better — Improving the Health of the American People. N Engl J Med.

[8] Osterberg L, Blaschke T (2005). Adherence to Medication. N Engl J Med.

[9] Cramer J A, Benedict A, Muszbek N, Keskinaslan A, Khan Z M (2008). The significance of compliance and persistence in the treatment of diabetes, hypertension and dyslipidaemia: a review. Int J Clin Pract.

[10] Trueman P, Taylor DG, Lowson K, Bligh A, Meszaros A, Wright D, Glanville J, Newbould J, Bury M, Barber N, Jani YH (2010). Evaluation of the scale, causes and costs of waste medicines: final report.

[11] Iuga Aurel O, McGuire Maura J (2014). Adherence and health care costs. Risk Manag Healthc Policy.

[12] Krapek K, King K, Warren SS, George KG, Caputo DA, Mihelich K, Holst EM, Nichol MB, Shi SG, Livengood KB, Walden S, Lubowski TJ (2004). Medication adherence and associated hemoglobin A1c in type 2 diabetes. Ann Pharmacother.

[13] Klein R (1995). Hyperglycemia and microvascular and macrovascular disease in diabetes. Diabetes Care.

[14] Pirart J (1978). Diabetes Mellitus and Its Degenerative Complications: A Prospective Study of 4,400 Patients Observed Between 1947 and 1973. Diabetes Care.

[15] Adler AI, Boyko EJ, Ahroni JH, Stensel V, Forsberg RC, Smith DG (1997). Risk Factors for Diabetic Peripheral Sensory Neuropathy: Results of the Seattle Prospective Diabetic Foot Study. Diabetes Care.

[16] Turner RC, Millns H, Neil HAW, Stratton IM, Manley SE, Matthews DR, Holman RR (1998). Risk factors for coronary artery disease in non-insulin dependent diabetes mellitus: United Kingdom prospective diabetes study (UKPDS: 23). BMJ.

[17] Kuusisto J, Mykkänen L, Pyörälä K, Laakso M (1994). NIDDM and Its Metabolic Control Predict Coronary Heart Disease in Elderly Subjects. Diabetes.

[18] Lehto S, Rönnemaa T, Pyörälä K, Laakso M (1996). Predictors of Stroke in Middle-Aged Patients With Non–Insulin-Dependent Diabetes. Stroke.

[19] Standl E, Balletshofer B, Dahl B, Weichenhain B, Stiegler H, Hörmann A, Holle R (1996). Predictors of 10-year macrovascular and overall mortality in patients with NIDDM: the Munich General Practitioner Project. Diabetologia.

[20] Groeneveld Y, Petri H, Hermans J, Springer MP (1999). Relationship between blood glucose level and mortality in Type 2 diabetes mellitus: a systematic review. Diabet Med.

[21] Uusitupa MIJ, Niskanen LK, Siitonen O, Voutilainen E, Pyörälä K (1993). Ten-year cardiovascular mortality in relation to risk factors and abnormalities in lipoprotein composition in type 2 (non-insulin-dependent) diabetic and non-diabetic subjects. Diabetologia.

[22] Wei M, Gaskill SP, Haffner SM, Stern MP (1998). Effects of Diabetes and Level of Glycemia on All-Cause and Cardiovascular Mortality: The San Antonio Heart Study. Diabetes Care.

[23] Hanefeld M, Fischer S, Julius U, Schulze J, Schwanebeck U, Schmechel H, Ziegelasch HJ, Lindner J (1996). Risk factors for myocardial infarction and death in newly detected NIDDM: the Diabetes Intervention Study, 11-year follow-up. Diabetologia.

[24] Knuiman MW, Welborn TA, Whittall DE (1992). An analysis of excess mortality rates for persons with non-insulin-dependent diabetes mellitus in Western Australia using the Cox proportional hazards regression model. Am J Epidemiol.

[25] Sasaki A, Uehara M, Horiuchi N, Hasagawa K (1983). A long-term follow-up study of Japanese diabetic patients: mortality and causes of death. Diabetologia.

[26] Stratton I M, Adler A I, Neil H A, Matthews D R, Manley S E, Cull C A, Hadden D, Turner R C, Holman R R (2000). Association of glycaemia with macrovascular and microvascular complications of type 2 diabetes (UKPDS 35): prospective observational study. BMJ.

[27] Rubin RR, Peyrot M (1999). Quality of life and diabetes. Diabetes Metab Res Rev.

[28] Ho PM, Rumsfeld JS, Masoudi FA, McClure DL, Plomondon ME, Steiner JF, Magid DJ (2006). Effect of Medication Nonadherence on Hospitalization and Mortality Among Patients With Diabetes Mellitus. Arch Intern Med.

[29] Young-Hyman D, de Groot M, Hill-Briggs F, Gonzalez J, Hood K, Peyrot M (2016). Psychosocial Care for People With Diabetes: A Position Statement of the American Diabetes Association. Diabetes Care.

[30] (2017). National Institute for Health and Care Excellence (NICE).

[31] Padwal R, Li SK, Lau DCW (2003). Long-term pharmacotherapy for overweight and obesity: a systematic review and meta-analysis of randomized controlled trials. Int J Obes Relat Metab Disord.

[32] Stevens VJ, Obarzanek E, Cook N R, Lee I M, Appel L J, Smith West D, Milas N C, Mattfeldt-Beman M, Belden L, Bragg C, Millstone M, Raczynski J, Brewer A, Singh B, Cohen J, Trials for the Hypertension Prevention Research Group (2001). Long-term weight loss and changes in blood pressure: results of the Trials of Hypertension Prevention, phase II. Ann Intern Med.

[33] Dombrowski SU, Knittle K, Avenell A, Araújo-Soares V, Sniehotta FF (2014). Long term maintenance of weight loss with non-surgical interventions in obese adults: systematic review and meta-analyses of randomised controlled trials. BMJ.

[34] Karlsson J, Taft C, Rydén A, Sjöström L, Sullivan M (2007). Ten-year trends in health-related quality of life after surgical and conventional treatment for severe obesity: the SOS intervention study. Int J Obes.

[35] Carpenter MJ, Jardin BF, Burris JL, Mathew AR, Schnoll RA, Rigotti NA, Cummings KM (2013). Clinical Strategies to Enhance the Efficacy of Nicotine Replacement Therapy for Smoking Cessation: A Review of the Literature. Drugs.

[36] Hughes J R, Keely J, Naud S (2004). Shape of the relapse curve and long-term abstinence among untreated smokers. Addiction.

[37] Moos RH, Moos BS (2006). Rates and predictors of relapse after natural and treated remission from alcohol use disorders. Addiction.

[38] Elfhag K, Rossner S (2005). Who succeeds in maintaining weight loss? A conceptual review of factors associated with weight loss maintenance and weight regain. Obesity Reviews.

[39] Kwasnicka D, Dombrowski SU, White M, Sniehotta F (2016). Theoretical explanations for maintenance of behaviour change: a systematic review of behaviour theories. Health Psychol Rev.

[40] Albarracín D, Gillette JC, Earl AN, Glasman LR, Durantini MR, Ho M (2005). A test of major assumptions about behavior change: a comprehensive look at the effects of passive and active HIV-prevention interventions since the beginning of the epidemic. Psychol Bull.

[41] Hobbs N, Godfrey A, Lara J, Errington L, Meyer TD, Rochester L, White M, Mathers JC, Sniehotta FF (2013). Are behavioral interventions effective in increasing physical activity at 12 to 36 months in adults aged 55 to 70 years? A systematic review and meta-analysis. BMC Med.

[42] Garnett CV, Crane D, Brown J, Kaner EFS, Beyer FR, Muirhead CR, Hickman M, Beard E, Redmore J, de Vocht F, Michie S (2018). Behavior Change Techniques Used in Digital Behavior Change Interventions to Reduce Excessive Alcohol Consumption: A Meta-regression. Ann Behav Med.

[43] Voils CI, Gierisch JM, Yancy WS, Sandelowski M, Smith R, Bolton J, Strauss JL (2014). Differentiating Behavior Initiation and Maintenance: Theoretical Framework and Proof of Concept. Health Educ Behav.

[44] Hardeman W, Johnston M, Johnston D, Bonetti D, Wareham N, Kinmonth AL (2002). Application of the Theory of Planned Behaviour in Behaviour Change Interventions: A Systematic Review. Psychology & Health.

[45] Panesar Arjun (2019). Machine Learning And AI For Healthcare: Big Data For Improved Health Outcomes.

[46] Saslow LR, Summers C, Aikens JE, Unwin DJ (2018). Outcomes of a Digitally Delivered Low-Carbohydrate Type 2 Diabetes Self-Management Program: 1-Year Results of a Single-Arm Longitudinal Study. JMIR Diabetes.

[47] Michie S, van Stralen MM, West R (2011). The behaviour change wheel: A new method for characterising and designing behaviour change interventions. Implementation Sci.

[48] Jackson C, Eliasson L, Barber N, Weinman JA (2014). Applying COM-B to medication adherence. Eur Health Psychol.

[49] Michie S, Abraham C, Eccles MP, Francis JJ, Hardeman W, Johnston M (2011). Strengthening evaluation and implementation by specifying components of behaviour change interventions: a study protocol. Implementation Sci.

[50] Cane J, O’Connor D, Michie S (2012). Validation of the theoretical domains framework for use in behaviour change and implementation research. Implementation Sci.

[51] Holt-Lunstad J (2018). Why Social Relationships Are Important for Physical Health: A Systems Approach to Understanding and Modifying Risk and Protection. Annu. Rev. Psychol.

[52] Levy RL (1983). Social support and compliance: A selective review and critique of treatment integrity and outcome measurement. Social Science & Medicine.

[53] Deakin T, McShane CE, Cade JE, Williams R (2005). Group based training for self-management strategies in people with type 2 diabetes mellitus. Cochrane Database Syst Rev.

[54] Trento M, Passera P, Tomalino M, Bajardi M, Pomero F, Allione A, Vaccari P, Molinatti GM, Porta M (2001). Group Visits Improve Metabolic Control in Type 2 Diabetes: A 2-year follow-up. Diabetes Care.

[55] Sani M, Makeen A, Albasheer OBA, Solan YMH, Mahfouz MS (2018). Effect of telemedicine messages integrated with peer group support on glycemic control in type 2 diabetics, Kingdom of Saudi Arabia. Int J Diabetes Dev Ctries.

[56] Keyserling TC, Samuel-Hodge CD, Ammerman AS, Ainsworth BE, Henríquez-Roldán Carlos F, Elasy TA, Skelly AH, Johnston LF, Bangdiwala SI (2002). A randomized trial of an intervention to improve self-care behaviors of African-American women with type 2 diabetes: impact on physical activity. Diabetes Care.

[57] Bernardi R, Wu PF (2017). The Impact of Online Health Communities on Patients' Health Self-Management.

[58] Barrera M, Glasgow RE, McKay H, Boles S, Feil E (2002). Do Internet-based support interventions change perceptions of social support?: An experimental trial of approaches for supporting diabetes self-management. Am J Community Psychol.

[59] Wing RR, Marcus MD, Epstein LH, Jawad A (1991). A "family-based" approach to the treatment of obese type II diabetic patients. J Consult Clin Psychol.

[60] Heisler M (2007). Overview of Peer Support Models to Improve Diabetes Self-Management and Clinical Outcomes. Diabetes Spectrum.

[61] Caro JF, Fisher EB (2010). A solution might be within people with diabetes themselves. Fam Pract.

[62] Turner-McGrievy GM, Tate DF (2013). Weight loss social support in 140 characters or less: use of an online social network in a remotely delivered weight loss intervention. Transl Behav Med.

[63] Teyhen DS, Aldag M, Centola D, Edinborough E, Ghannadian JD, Haught A, Jackson T, Kinn J, Kunkler KJ, Levine B, Martindale VE, Neal D, Snyder LB, Styn MA, Thorndike F, Trabosh V, Parramore DJ (2014). Key enablers to facilitate healthy behavior change: workshop summary. J Orthop Sports Phys Ther.

[64] Sylvetsky AC, Nandagopal Radha, Nguyen Tammy T, Abegg Marisa R, Nagarur Mahathi, Kaplowitz Paul, Rother Kristina I (2015). Buddy Study: Partners for better health in adolescents with type 2 diabetes. World J Diabetes.

[65] Greaney ML, Puleo E, Sprunck-Harrild K, Haines J, Houghton SC, Emmons KM (2018). Social Support for Changing Multiple Behaviors: Factors Associated With Seeking Support and the Impact of Offered Support. Health Educ Behav.

[66] Sheldon KM, Kasser T, Smith K, Share T (2002). Personal Goals and Psychological Growth: Testing an Intervention to Enhance Goal Attainment and Personality Integration. J Personality.

[67] Burgess E, Hassmén P, Welvaert M, Pumpa K (2017). Behavioural treatment strategies improve adherence to lifestyle intervention programmes in adults with obesity: a systematic review and meta-analysis. Clin Obes.

[68] De Shazer S (1978). Brief hypnotherapy of two sexual dysfunctions: the crystal ball technique. Am J Clin Hypn.

[69] Gingerich WJ, Peterson LT (2013). Effectiveness of Solution-Focused Brief Therapy. Research on Social Work Practice.

[70] Viner R M, Christie D, Taylor V, Hey S (2003). Motivational/solution-focused intervention improves HbA1c in adolescents with Type 1 diabetes: a pilot study. Diabet Med.

[71] Locke E, Latham GP (1990). A theory of goal setting & task performance.

[72] Amireault S, Godin G, Vézina-Im L (2013). Determinants of physical activity maintenance: a systematic review and meta-analyses. Health Psychology Review.

[73] West DS, Gorin AA, Subak LL, Foster G, Bragg C, Hecht J, Schembri M, Wing RR (2010). A motivation-focused weight loss maintenance program is an effective alternative to a skill-based approach. Int J Obes.

[74] Strecher VJ, Seijts GH, Kok GJ, Latham GP, Glasgow R, DeVellis B, Meertens RM, Bulger DW (1995). Goal setting as a strategy for health behavior change. Health Educ Q.

[75] Harkin B, Webb TL, Chang BPI, Prestwich A, Conner M, Kellar I, Benn Y, Sheeran P (2016). Does monitoring goal progress promote goal attainment? A meta-analysis of the experimental evidence. Psychol Bull.

[76] Chung C, Cook J, Bales E, Zia J, Munson SA (2015). More Than Telemonitoring: Health Provider Use and Nonuse of Life-Log Data in Irritable Bowel Syndrome and Weight Management. J Med Internet Res.

[77] Gadkari AS, McHorney CA (2012). Unintentional non-adherence to chronic prescription medications: How unintentional is it really?. BMC Health Serv Res.

[78] Osterberg L, Blaschke T (2005). Adherence to Medication. N Engl J Med.

[79] Wroe A (2002). Intentional and Unintentional Nonadherence: A Study of Decision Making. J Behav Med.

[80] Lawrence DB, Allison W, Chen JC, Demand M (2008). Improving Medication Adherence with a Targeted, Technology-Driven Disease Management Intervention. Disease Management.

[81] Walker EA, Shmukler C, Ullman R, Blanco E, Scollan-Koliopoulus M, Cohen HW (2010). Results of a Successful Telephonic Intervention to Improve Diabetes Control in Urban Adults: A randomized trial. Diabetes Care.

[82] Kamal AK, Shaikh Q, Pasha O, Azam I, Islam M, Memon AA, Rehman H, Akram MA, Affan M, Nazir S, Aziz S, Jan M, Andani A, Muqeet A, Ahmed B, Khoja S (2015). A randomized controlled behavioral intervention trial to improve medication adherence in adult stroke patients with prescription tailored Short Messaging Service (SMS)-SMS4Stroke study. BMC Neurol.

[83] Guy R, Hocking J, Wand H, Stott S, Ali H, Kaldor J (2011). How Effective Are Short Message Service Reminders at Increasing Clinic Attendance? A Meta-Analysis and Systematic Review. Health Serv Res.

[84] Robotham D, Satkunanathan S, Reynolds J, Stahl D, Wykes T (2016). Using digital notifications to improve attendance in clinic: systematic review and meta-analysis. BMJ Open.

[85] Franklin VL, Waller A, Pagliari C, Greene SA (2006). A randomized controlled trial of Sweet Talk, a text-messaging system to support young people with diabetes. Diabetic Med.

[86] Patrick K, Raab F, Adams MA, Dillon L, Zabinski M, Rock CL, Griswold WG, Norman GJ (2009). A Text Message–Based Intervention for Weight Loss: Randomized Controlled Trial. J Med Internet Res.

[87] Locke EA (1968). Toward a theory of task motivation and incentives. Organizational Behavior and Human Performance.

[88] Brug J, de Vet E, de Nooijer J, Verplanken B (2006). Predicting fruit consumption: cognitions, intention, and habits. J Nutr Educ Behav.

[89] Brug J, Steenhuis I, van Assema P, de Vries H (1996). The impact of a computer-tailored nutrition intervention. Prev Med.

[90] Skinner CS, Strecher VJ, Hospers H (1994). Physicians' recommendations for mammography: do tailored messages make a difference?. Am J Public Health.

[91] Kreuter MW, Strecher VJ, Glassman B (1999). One size does not fit all: The case for tailoring print materials. Ann Behav Med.

[92] Kroeze W, Werkman A, Brug J (2006). A systematic review of randomized trials on the effectiveness of computer-tailored education on physical activity and dietary behaviors. Ann Behav Med.

[93] Lagisetty PA, Priyadarshini S, Terrell S, Hamati M, Landgraf J, Chopra V, Heisler M (2017). Culturally Targeted Strategies for Diabetes Prevention in Minority Population. Diabetes Educ.

[94] Kim MT, Kim KB, Huh B, Nguyen T, Han H, Bone LR, Levine D (2015). The Effect of a Community-Based Self-Help Intervention: Korean Americans With Type 2 Diabetes. Am J Prev Med.

[95] Hawthorne K, Robles Y, Cannings-John R, Edwards A G K (2010). Culturally appropriate health education for Type 2 diabetes in ethnic minority groups: a systematic and narrative review of randomized controlled trials. Diabet Med.

[96] Clark M, Hampson SE, Avery L, Simpson R (2004). Effects of a tailored lifestyle self-management intervention in patients with type 2 diabetes. Br J Health Psychol.

[97] Liu J, Davidson E, Bhopal R, White M, Johnson M, Netto G, Deverill M, Sheikh A (2012). Adapting health promotion interventions to meet the needs of ethnic minority groups: mixed-methods evidence synthesis. Health Technol Assess.

[98] Kreuter MW, Wray RJ (2003). Tailored and targeted health communication: strategies for enhancing information relevance. Am J Health Behav.

[99] David SK, Rafiullah M (2017). Innovative health informatics as an effective modern strategy in diabetes management: a critical review. Case Study of Innovative Projects - Successful Real Cases.

[100] Burner E, Lam CN, DeRoss R, Kagawa-Singer M, Menchine M, Arora S (2018). Using Mobile Health to Improve Social Support for Low-Income Latino Patients with Diabetes: A Mixed-Methods Analysis of the Feasibility Trial of TExT-MED + FANS. Diabetes Technol Ther.

[101] Butt S, Navarro KF, Shorab M, Onn A (2016). Using Mobile Technology to Improve Nutritional Information of Diabetic Patients. New Advances in Information Systems and Technologies. Advances in Intelligent Systems and Computing.

[102] Pillay J, Armstrong MJ, Butalia S, Donovan LE, Sigal RJ, Vandermeer B, Chordiya P, Dhakal S, Hartling L, Nuspl M, Featherstone R, Dryden DM (2015). Behavioral Programs for Type 2 Diabetes Mellitus: A Systematic Review and Network Meta-analysis. Ann Intern Med.

[103] Bolier L, Haverman M, Kramer J, Boon B, Smit F, Riper H, Bohlmeijer E (2012). Internet-Based Intervention to Promote Mental Fitness in Mildly Depressed Adults: Design of a Randomized Controlled Trial. JMIR Res Protoc.

[104] Spek V, Cuijpers P, Nyklícek I, Riper H, Keyzer J, Pop V (2007). Internet-based cognitive behaviour therapy for symptoms of depression and anxiety: a meta-analysis. Psychol Med.

[105] Murray E, Hekler EB, Andersson G, Collins LM, Doherty A, Hollis C, Rivera DE, West R, Wyatt JC (2016). Evaluating Digital Health Interventions: Key Questions and Approaches. Am J Prev Med.

[106] Faruque LI, Wiebe N, Ehteshami-Afshar A, Liu Y, Dianati-Maleki N, Hemmelgarn BR, Manns BJ, Tonelli M, Alberta Kidney Disease Network (2017). Effect of telemedicine on glycated hemoglobin in diabetes: a systematic review and meta-analysis of randomized trials. CMAJ.

[107] Bonoto BC, de Araújo VE, Godói IP, de Lemos LLP, Godman B, Bennie M, Diniz LM, Junior AAG (2017). Efficacy of Mobile Apps to Support the Care of Patients With Diabetes Mellitus: A Systematic Review and Meta-Analysis of Randomized Controlled Trials. JMIR Mhealth Uhealth.

[108] Levy MD, Loy L, Zatz LY (2014). Policy approach to nutrition and physical activity education in health care professional training. Am J Clin Nutr.

[109] Campbell D The Guardian.

