# Supplementary figures and images for: Novel Digital Architecture of a “Low Carb Program” for Initiating and Maintaining Long-Term Sustainable Health-Promoting Behavior Change in Patients with Type 2 Diabetes
Source: JMIR Diabetes. 2020 Mar 4;5(1):e15030. doi: 10.2196/15030 (PMC7081139; doi:10.2196/15030)

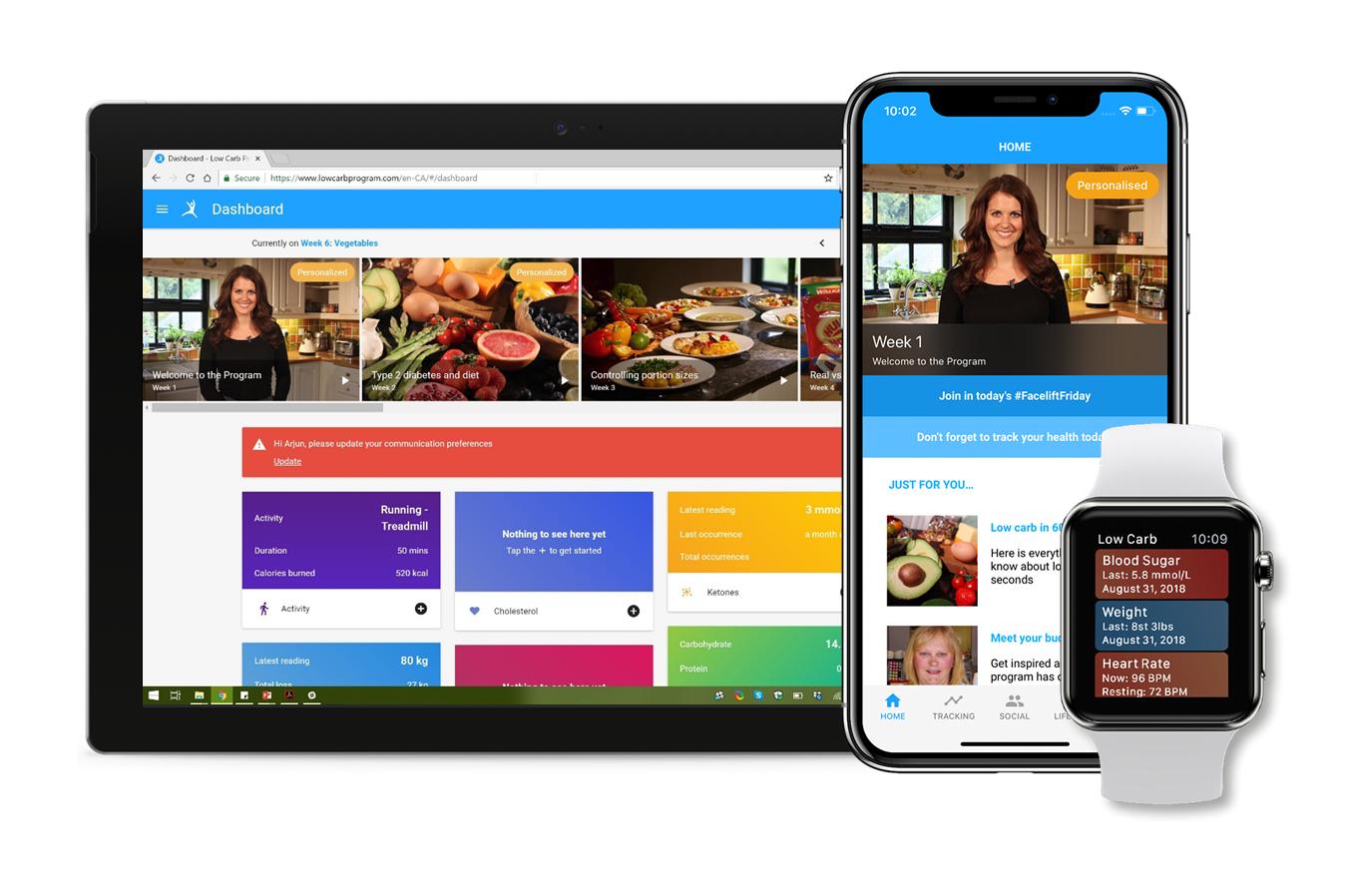

Supplement: Multimedia Appendix 1 [file diabetes_v5i1e15030_app1.png]

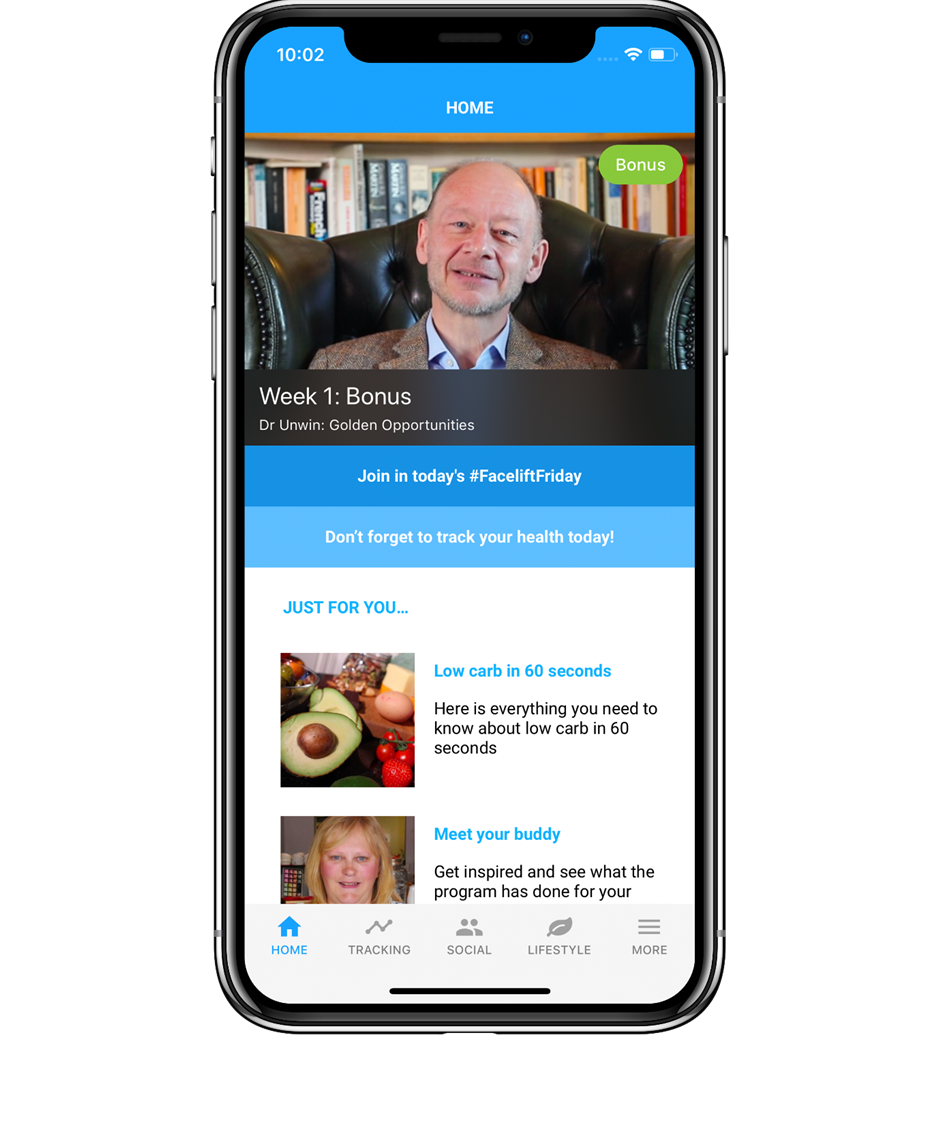

Supplement: Multimedia Appendix 2 [file diabetes_v5i1e15030_app2.png]
